# A Comparison of Intralesional Triamcinolone Acetonide Injection for Primary Chalazion in Children and Adults

**DOI:** 10.1155/2014/413729

**Published:** 2014-10-15

**Authors:** Jacky W. Y. Lee, Gordon S. K. Yau, Michelle Y. Y. Wong, Can Y. F. Yuen

**Affiliations:** Department of Ophthalmology, Caritas Medical Centre, 111 Wing Hong Street, Kowloon, Hong Kong Special Administrative Region, Hong Kong

## Abstract

*Purpose.* To investigate outcome differences of intralesional triamcinolone acetonide (TA) injection for primary chalazia in children versus adults.* Methods*. A retrospective review of consecutive subjects with primary chalazion who received intralesional TA injection was conducted. A single investigator injected 0.05–0.15 mL of TA (40 mg/mL) intralesionally. Patients were stratified into the pediatric (<18 years old) and adult (≥18 years old) group. In both groups, the correlation of resolution time with chalazion size and TA dose was performed.* Results*. 17 children and 24 adults were enrolled, with a mean age of 7.4 ± 5.5 and 39.3 ± 16.7 years, respectively. Both groups had statistically similar baseline characteristics. There was no significant difference between the resolution time in the pediatric (18.2 ± 11.4 days) and adult (16.5 ± 11.0 days) group (*P* = 0.7). There were no significant complications from the TA injection. There was no significant correlation of resolution time to chalazion size (*P* = 0.7) nor TA dose (*P* = 0.3) in both groups.* Conclusion*. TA for the treatment of primary chalazion was equally effective in children and adults, without any significant complications, and the rate of clinical response did not appear to be dose-dependent.

## 1. Introduction

Chalazion is a localized chronic granulomatous inflammation following blockage of the meibomian glands, more commonly affecting the upper eyelids. The range of presentation can be from a benign, self-limiting nodule to a painful lid swelling complicated by corneal astigmatism and mechanical ptosis from the space-occupying effect of the chalazion in the relatively limited eyelid space [1]. Chalazia are initially managed conservatively using warm compress and antibiotic eye ointment for the prevention of secondary bacterial infection. For persistent lesions, incision and curettage (I&C), steroid injection, or carbon dioxide laser treatment may be considered [2, 3]. I&C warrants referral to an ophthalmologist which takes time and may be associated with surgical risks including pain, bleeding, and scarring. Intralesional steroid injection for chalazion has been reported to be effective for the treatment of chalazia with high success rates [2–10]. This treatment modality is particularly useful in children and in patients where cooperation for I&C is difficult as the procedure involved is equivalent to the injection of local anesthesia required for I&C.

The aim of this study was to investigate the differences in outcome of using intralesional triamcinolone acetonide (TA) injection for the treatment of primary chalazia in children and adults.

## 2. Patients and Methods

Ethics approval by the Institution Review Board was obtained and the study adhered to the Declaration of Helsinki. The authors declare no financial or proprietary interest. This was a retrospective case series from a district hospital in Hong Kong Special Administrative Region, China, with a service population of 1.8 million. Patient medical records from January 2012 to March 2013 were reviewed for all subjects that underwent intralesional TA injection for primary chalazion not responding to conservative treatment. All injections were done by a single ophthalmologist (SKY). The inclusion criteria included consecutive subjects with the diagnosis of chalazion who consented for intralesional TA injection after failure of conservative treatment with lid hygiene, warm compression, and antibiotic ointment for at least 1 month. The exclusion criteria included those with eyelid infection, chalazion duration < 1 month, nonpalpable chalazion, suspicion of malignancy, a history of steroid induced elevated intraocular pressure (IOP), or those that defaulted follow-up after the injection. Informed consent was obtained before the procedure was carried out from the patient or the patient's legal guardian for those <18 years of age.

The outcome measures included chalazion size (length × width) in millimetres (mm), dose of TA injected, time to complete resolution of the chalazion, and complications from the procedure.

### 2.1. Technique of Triamcinolone Injection

Topical anaesthesia (proparacaine 0.5%) eye drops were instilled in the affected eye before the injection. A volume of 0.05 to 0.15 mL of TA (40 mg/mL) (Stacort-A, Standard Chem & Pharm Co., Ltd., No. 6-20, Tuku., Tuku Village, Sinying District, Tainan City 73055, Taiwan) was injected intralesionally in the out-patient treatment room according to the maximal diameter of the chalazion as follows: <1 cm = 2 mg/0.05 mL TA; 1–1.5 cm = 4 mg/0.1 mL TA; and >1.5 cm = 6 mg/0.15 mL TA. The eyelid was inverted and the TA was injected transconjunctivally into the centre of the lesion with a 27-gauge needle. When it was not possible to evert the eyelid due to extensive swelling, the injection was given transcutaneously into the chalazion after disinfection of the skin with 70% isopropyl alcohol wipes. No patching was required after the procedure. The patients were given chloramphenicol 1% eye ointment three times per day to apply over the lesion and advised to continue warm compression for 4 to 6 times per day for 10 minutes each with a hard-boiled egg. The patients were reviewed every 2 weeks after the TA injection until complete resolution of the chalazion. For uncooperative or very young children, sedation was with oral chloral hydrate (50 mg/kg) was given 30 minutes before the procedure.

### 2.2. Statistics

Patients were stratified by age: the pediatric group (<18 years old) and adult group (≥18 years old). The following were analyzed for differences between pediatric and adult groups using the Mann Whitney *U* test: age, sex, laterality, mean chalazion size (length × width), TA dose, and time to resolution of the chalazion.

In both the pediatric and adult groups, correlation of time to resolution with chalazion size and TA dose was analyzed using the Spearman's rank correlation coefficient. All means were expressed as means ± standard deviation. Statistical significance was defined as *P* < 0.05.

## 3. Results

The mean age in the pediatric and adult group was 7.4 ± 5.5 and 39.3 ± 16.7 years old, respectively. Both the pediatric (17) and adult (24) groups had statistically similar baseline characteristics in terms of sex, laterality, mean chalazion size, and TA dose (Table 1). All patients were of Chinese ethnicity. There was no significant difference between the time taken for complete resolution of the chalazion between the pediatric (18.2 ± 11.4 days) and adult (16.5 ± 11.0 days) groups (*P* = 0.7) (Table 1). There were no significant complications from the TA injection in both groups.

There was no significant correlation of time to resolution with either chalazion size (*P* = 0.7) nor TA dose (*P* = 0.3) in both the pediatric and adult group (Table 2).

## 4. Discussion

Chalazion is a common cause of lid inflammation and is self-limiting with conservative warm compress in 29–80% [2, 4, 11, 12]. For persistent lesions, I&C and intralesional steroid injection are the most common procedures with reported success rates of 87–89% and 62–92%, respectively [2–10, 13, 14]. Whilst I&C seems to offer a more consistent success rate, intralesional steroid injection has the potential advantages of not requiring additional anesthetic injection, less bleeding, and scarring risk, can be performed in the office-setting, and may be used for multiple chalazia and even for lesions that are close to the lacrimal punctum and of course for those where cooperation is compromised like in children or adults with mental incapacities, dementia, or anxiety.

In our study, the pediatric and adult study group had statistically similar baseline characteristics apart from age. Despite the age differences and hence, size of the eyelids, both the pediatric and adult populations presented with a mean chalazion size of around 0.8 mm^2^ and subsequently received a similar dose (around 3 mg) of TA injection. TA injection was equally effective in both the pediatric and adult populations with a statistically similar recovery rate of a little more than 2 weeks in both groups (*P* = 0.7). Our findings are consistent with that of Pavicic-Astalos et al. [7] who reported a resolution time of 15.27 days after 4 to 8 mg of intralesional TA injection. Most importantly, there was no adverse outcome from the injection in both the pediatric and adult groups.

Palva and Pohjanpelto [2] reported that larger chalazia involved a slower recovery and a higher recurrence rate. In our study, we noted that the time taken for chalazion resolution was not significantly correlated with the chalazion size (*P* = 0.7) nor the amount of TA injected (*P* = 0.3), suggesting that the response to steroid injection may be independent of the lesion size and may not be dose-dependent.

Whilst TA injection is a simple and effective treatment for chalazion in both children and adults, it is important for clinicians to recognize the conditions in which TA injection should not be performed. A hordeolum can sometimes mimic a chalazion as it is a meibomian gland obstruction with a superimposed infection, usually* Staphylococcus aureus*, giving rise to a pustular swelling. The infective component of a hordeolum usually resolves in 1 week with topical antibiotics and may develop into a chalazion subsequently [15]. TA injection should not be given for hordeolums given its infective nature and likewise examination for preexisting follicles in the fornix under the slit lamp is important to rule out previous herpetic infections. If doubt exists in the differentiation, referral to an ophthalmologist is recommended.

For those presenting with recurrent chalazia in the same location, a high index of suspicion for sebaceous cell carcinoma should exist and biopsy and histological studies are indicated [16, 17].

Whilst none of the 41 subjects in our series had complications from the TA injection, clinicians carrying out such procedure should be aware of the potential complications including yellowish deposits at the injection site, elevated IOP, and skin hypopigmentations, globe perforation, traumatic cataract, microembolization, and retinal/choroidal vascular occlusions [18–22].

Our study was limited by its retrospective nature, relatively small sample size, and the lack of a control group to compare with other treatment modalities. Nevertheless, this study served its purpose in addressing that a single injection of intralesional TA for the treatment of primary chalazion was equally effective in children and adults, without any significant complications, and the rate of clinical response did not appear to be dose-dependent.

## Figures and Tables

**Table 1:** Differences in baseline and outcome in children versus adults.

	Pediatric (*n* = 17)	Adult (*n* = 24)	*P* value
Mean age (years)	7.4 ± 5.5	39.3 ± 16.7	<0.0001∗
Sex (M/F)	8/10	15/9	0.3
Laterality (R/L)	11/16	14/10	0.7
Mean chalazion size: length × width (mm^2^)	0.8. ± 0.5	0.8. ± 0.5	0.9
Mean TA dose (mg)	3.5 ± 1.3	3.2 ± 1.1	0.4
Time to resolution (days)	18.2 ± 11.4	16.5 ± 11.0	0.7

^*^Statistically significant.

**Table 2:** Correlations of TA dose and chalazion size with time to resolution in adults and children.

	Children (*n* = 17)	Adults (*n* = 24)
Correlation of time to resolution with TA dose [Spearman *r*/(*P* value)]	*r* = −0.1 (0.6)	*r* = 0.2 (0.3)
Correlation of time to resolution with chalazion size [Spearman *r*/(*P* value)]	*r* = −0.2 (0.4)	*r* = 0.06 (0.7)
